# Surgical strategy for intraductal papillary mucinous neoplasms of the pancreas

**DOI:** 10.1007/s00595-019-01931-5

**Published:** 2019-12-05

**Authors:** Seiko Hirono, Hiroki Yamaue

**Affiliations:** Second Department of Surgery, School of Medicine, Wakayama Medical University, 811-1 Kimiidera, Wakayama, 641-8510 Japan

**Keywords:** IPMN, CEA in the pancreatic juice, Mural nodule, High-grade dysplasia, Invasive IPMC

## Abstract

The current treatment strategy for intraductal papillary mucinous neoplasms (IPMNs), based on the international consensus guideline, has been accepted widely. However, reported outcomes after surgical resection for IPMN show that once the tumor progresses to invasive intraductal papillary mucinous carcinoma (IPMC), recurrence is not uncommon. The surgical treatment for IPMN is invasive and sometimes followed by complications. Therefore, the best timing for resection might be at the point when high-grade dysplasia (HGD) is evident. According to previous reports, main duct type IPMN has a high malignant potential and its surgical resection is universally accepted, whereas, the incidence of HGD/invasive IPMC in branch duct and mixed type IPMNs is thought to be lower. In addition to mural nodules and a dilated main pancreatic duct, cytology and measurement of the carcinoembryonic antigen level in the pancreatic juice might be useful to differentiate HGD/invasive IPMC from low-grade dysplasia. The nomogram proposed recently to predict the risk of HGD/invasive IPMC in IPMN patients might help surgeons decide on the best treatment strategy, depending on the patient’s age and general condition. Second resection for high-risk lesions in the remnant pancreas might improve the survival of IPMN patients.

## Introduction

The incidence of intraductal papillary mucinous neoplasm (IPMN) of the pancreas has increased recently in line with awareness and advances in radiographic and endoscopic imaging. IPMNs exhibit a spectrum of neoplastic transformation, classified as low-grade dysplasia (LGD), high-grade dysplasia (HGD), and invasive intraductal papillary carcinoma (IPMC) [1, 2]. The following three guidelines for the diagnosis and management of IPMNs have been used widely: The international consensus guideline (ICG) [1, 3, 4], the European evidence-based guideline [5], and the American Gastroenterological Association (AGA) Institute guideline [6]. There appears to be a recent trend toward an increase in the number of IPMN patients being followed up but not undergoing surgical resection for three reasons: First, the number of patients with IPMN diagnosed early is increasing with advances in radiographic and endoscopic imaging [7–10]; second, the operative treatment is invasive; and third, the frequency of postoperative complications after pancreatectomy is higher than that after most other gastrointestinal operations. However, in some patients, IPMN may become invasive during follow-up, and recurrence can develop even after surgery, with progression to unresectable invasive IPMC from metastases or local spread. To avoid these miserable outcomes, it would be advantageous to identify when IPMN should be resected and which factors are significantly associated with the appropriate surgical indications for IPMN.

Recent studies have demonstrated the malignant progression of IPMN and that pancreatic ductal adenocarcinoma (PDAC), being a high-risk lesion, may develop in the remnant pancreas after surgical resection for IPMN [11–15]. It is important that we evaluate what treatment could improve the survival of these patients with high-risk lesions in the remnant pancreas after surgery for IPMN. In this article, we review which patients would gain oncological benefits from surgical resection for IPMN and what operative procedures are appropriate.

### Surgical indication of IPMN based on outcomes after resection

According to previous reports, the frequency of overall recurrence after surgical resection for noninvasive IPMN (LGD/HGD) ranges from 0 to 17% [11, 12, 15–17]. In a Japanese multicenter study of 1074 IPMN patients who underwent surgery, recurrence developed after surgery in 0.7% of 827 patients with LGD/HGD, but in 43.3% of 247 patients with invasive IPMC [11]. Thus, once the tumor progresses to having an invasive component, recurrence is likely, even after surgery [11, 12, 18]. However, pancreatectomy for IPMN is invasive, despite advanced and optimally safe operative techniques. Therefore, if a tumor is diagnosed as LGD IPMN by radiographic and endoscopic findings, it could be monitored without surgery. In light of these results, HGD might be the best timing for surgical resection for IPMN.

The revised ICGs in 2012 and 2017 defined that malignant IPMN includes only invasive IPMC, but not HGD [1, 3], and this definition could confuse clinicians about the surgical indication, HGD and invasive IPMC (HGD/invasive IPMC), or only invasive IPMC. In consideration of the poor prognosis after surgery for invasive IPMC, we believe that HGD/invasive IPMC is the surgical indication, and it is important to identify the predictive factors associated with HGD/invasive IPMC.

### Predictive factors associated with HGD/invasive IPMC

Depending on the morphology of the ductal system, IPMNs are classified into three types: main duct (MD) type, branch duct (BD) type, and mixed type [1, 3–5]. In MD type IPMNs, the frequency of HGD/invasive IPMC ranges from 36 to 100% and the frequency of invasive IPMC ranges from 11 to 81% [16, 19–28]. Considering these high incidences of HGD/invasive IPMC, surgical resection for MD type IPMN is universally accepted. Conversely, in BD/mixed-type IPMNs, the incidence of HGD/invasive IPMC ranges from 14.4 to 47.9%, and the incidence of invasive IPMC ranges from 6.1 to 37.7% [29–35]. Thus, the frequency of HGD/invasive IPMC in BD/mixed-type IPMNs is significantly lower than those in MD type IPMN, and we should consider the predictive factors associated with this.

The ICG revised in 2017 described three factors as high-risk stigmata; namely, obstructive jaundice, enhancing of a mural nodule ≥ 5 mm, and a main pancreatic duct (MPD) ≥ 10 mm; and that any of these high-risk factors should be an absolute surgical indication [1]. Furthermore, endoscopic ultrasonography (EUS) is recommended for patients with any of the following nine features of concern: pancreatitis, cyst ≥ 3 cm, enhancing mural nodule < 5 mm, thickened/enhancing cyst wall, MPD 5–9 mm, abrupt change in caliber or pancreatic duct with distal pancreatic atrophy, lymphadenopathy, increased serum carbohydrate antigen 19-9 (CA19-9) level, and a cyst growth rate ≥ 5 mm/2 years. Subsequently, any of the three following findings by EUS would define surgical indication: mural nodule ≥ 5 mm, MPD features suspicious for involvement, and positive cytology [1].

The European guideline suggested that the absolute indications for surgery are positive cytology, a solid mass, tumor-related jaundice, enhancing mural nodules ≥ 5 mm, and MPD ≥ 10 mm. These factors are similar to the high-risk stigmata in ICG. This guideline also suggests the following relative indications: a growth rate ≥ 5 mm/year, an increased serum CA19-9 level, MPD 5-9.9 mm, cyst diameter ≥ 40 mm, onset of diabetes mellitus, acute pancreatitis caused by IPMN, and enhancing mural nodules < 5 mm [5]. It recommends surgery for patients without significant co-morbidities and one or more relative indication for surgery and for patients with significant co-morbidities and two or more relative indications for surgery [5].

The AGA guideline suggests that both a solid component and a dilated MPD and/or concerning features on EUS and FNA are indications for surgery [6]. According to these guidelines, which are based on evidence from many reports, mural nodule and dilated MPD on radiographic image, and obstructive jaundice or pancreatitis caused by IPMN are important indications for surgery.

In addition to a mural nodule and dilated MPD, several studies have found that cytology and/or tumor markers, including carcinoembryonic antigen (CEA) in the cystic fluid obtained by EUS-guided fine needle aspiration (EUS-FNA), or in the pancreatic juice obtained from MPD by endoscopic retrograde pancreatography (ERP), were useful to establish surgical indication for IPMN [36–41]. The diagnostic accuracy of cystic fluid or pancreatic juice cytology for HGD/invasive IPMC is poor because the sensitivity rate is low [36–38]. However, the positive predictive value (PPV) of cytology for HGD/invasive IPMC is excellent and might be a useful method to determine surgical indication [36, 37]. Several studies have reported the usefulness of measuring the CEA level in pancreatic juice obtained from MPD by ERP to differentiate LGD from HGD/invasive IPMC [18, 38–40]. However, there are few reports documenting the usefulness of measuring the CEA level in cystic fluid obtained by EUS-FNA [41]. We consider that BD-type IPMNs often consist of multilocular cysts, but it is unknown which cyst has the most severe atypia, whereas the pancreatic juice in the MPD obtained by ERP included secreted CEA from all of the pancreatic ducts. That is why measurement of the CEA level in pancreatic juice might be more useful than measuring that in the cystic fluid. Furthermore, EUS-FNA for IPMN carries a theoretical risk of peritoneal seeding, and there have been several reports of this [42, 43], whereas obtaining pancreatic juice by ERP is not associated with a risk of peritoneal seeding. Nevertheless, the ICG do not recommend routine ERP for collecting pancreatic juice during evaluation of the malignant potential of an IPMN because the procedure is invasive and could cause post-ERP pancreatitis [1, 3]. Based on these findings, evaluation of the cytology or measurement of the CEA level in pancreatic juice obtained by ERP is helpful for determining the surgical indication for IPMN, whereas because ERP is an invasive procedure, it should be performed only as a diagnostic adjunct, not as a routine examination.

Recent large-scale studies have proposed using a nomogram to predict the individual risk of HGD/invasive IPMC in patients with IPMN [44–47]. These models show the diagnostic ability of HGD/invasive IPMC, and are contributory to determining the best treatment strategy, depending on the patient’s age and general condition.

### Treatment strategy for invasive IPMC

According to a Japanese multicenter study of 1074 IPMN patients, 30.8% of 247 patients with invasive IPMC treated by surgery had lymph node metastases, including 2.7% of 74 with T1a invasive IPMC with a depth of invasive component of ≤ 5 mm [11]. This is consistent with other reports [12, 15, 16]. Based on these findings, lymph node dissection same as that done for PDAC might be necessary for pancreatectomy for invasive IPMC to improve survival. Therefore, it is important to identify the predictive factors associated with invasive IPMC to decide on the most appropriate operative procedure. We reported previously that a large mural nodule was an independent predictor of invasive IPMC in all types of IPMNs (MD, BD, and mixed types), and a high CEA level in the pancreatic juice was an independent factor of invasive IPMC in MD and mixed-type IPMNs [48]. The diagnostic accuracy using our predictors was significantly higher than that using high-risk stigmata or features of concern in the ICG [3, 48]. Evaluation of mural nodule size and CEA level in the pancreatic juice might be useful to predict the appropriate degree of lymph node dissection during pancreatectomy, preoperatively.

The oncological benefits of postoperative adjuvant therapy for invasive IPMC remain controversial. Some studies have found that postoperative adjuvant therapy does not impact on the survival of patients with invasive IPMC [11, 49]. Other studies have found that postoperative adjuvant therapy could improve the survival of patients with invasive IPMC patients with advanced stage or lymph node metastases [50, 51]. To confirm the oncological benefits of postoperative adjuvant therapy for invasive IPMC patients, a large-scale prospective study is necessary.

### Optimal operative procedures for noninvasive IPMN

Patients with noninvasive IPMN are expected to survive long-term; therefore, quality of life, including endocrine and exocrine function of the remnant pancreas after surgery, must be considered. Several studies have reported the feasibility of IPMN enucleation, but this is controversial [52–54]. While tumor enucleation can preserve most pancreatic parenchyma, resulting in long-term good pancreatic function, performing this procedure for IPMN carries a risk of rupturing the cysts during the operation, causing peritoneal seeding. Moreover, it is frequently associated with postoperative complications including pancreatic fistula.

For noninvasive IPMN located in the pancreatic body, several investigators have reported better long-term outcomes after central pancreatectomy than after distal pancreatectomy [55–57]. Furthermore, recent reports have shown that laparoscopic or robotic pancreatectomy for IPMN is feasible and minimally invasive [58–60]. Establishing an appropriate operative procedure for noninvasive IPMN from the viewpoint not only of short-term outcomes, including postoperative complications, but also of long-term outcomes, including preserving function of the remnant pancreas, is critical.

### Treatment strategy for high-risk lesions in the remnant pancreas after surgical resection for IPMN

According to the Japanese multicenter study of 1074 IPMN patients, the 5-year and 10-year cumulative incidences of high-risk lesions; namely, malignant progression of IPMN and new development of PDAC, in the remnant pancreas after surgical resection for IPMN, were 6.2% and 12.6%, respectively [11]. Furthermore, second surgery for metachronous high-risk lesions in the remnant pancreas seemed to improve survival [11]. Based on these results, lifelong surveillance of the remnant pancreas following surgery might be necessary for IPMN patients, to enable us to diagnose and treat high-risk lesions in the remnant pancreas at an early stage.

## Conclusions

To improve the survival of IPMN patients, resection might be necessary before the tumor progresses to having invasive components. Evaluation of the mural nodule and MPD size, in addition to symptoms including obstructive jaundice and pancreatitis, will help to determine the surgical indication for IPMN. Furthermore, measurement of the CEA level in the pancreatic juice might be useful to determine the surgical indication and the most appropriate operative procedure. High-risk lesions in the remnant pancreas, such as malignant progression of IPMN or new development of PDAC, could develop over 5 years, and then lifelong surveillance after surgery is necessary. Second surgery for high-risk lesions in the remnant pancreas might also improve survival.
